# dDAVP Downregulates the AQP3-Mediated Glycerol Transport *via* V1aR in Human Colon HCT8 Cells

**DOI:** 10.3389/fcell.2022.919438

**Published:** 2022-07-08

**Authors:** Mariangela Centrone, Mariagrazia D’Agostino, Marianna Ranieri, Maria Grazia Mola, Pinuccia Faviana, Piero Vincenzo Lippolis, Domenico Alessandro Silvestris, Maria Venneri, Annarita Di Mise, Giovanna Valenti, Grazia Tamma

**Affiliations:** ^1^ Department of Biosciences, Biotechnologies and Biopharmaceutics, University of Bari Aldo Moro, Bari, Italy; ^2^ Department of Surgical, Medical, Molecular Pathology, and Critical Area, University of Pisa, Pisa, Italy; ^3^ Department of Surgery, University of Pisa, Pisa, Italy; ^4^ Department of Onco-haematology, IRCCS Ospedale Pediatrico Bambino Gesù, Rome, Italy

**Keywords:** vasopressin, V1aR, V2R, AQP3, glycerol, tolvaptan, SR49059, DFP00173

## Abstract

Vasopressin (AVP) plays a key function in controlling body water and salt balance through the activation of the vasopressin receptors V1aR and V2R. Abnormal secretion of AVP can cause the syndrome of inappropriate antidiuresis that leads to hyponatremia, which is an electrolyte disorder often observed in the elderly hospitalized and oncologic patients. Beyond kidneys, the colonic epithelium modulates water and salt homeostasis. The water channel AQP3, expressed in villus epithelial cells is implicated in water absorption across human colonic surface cells. Here, the action of dDAVP, a stable vasopressin analog, was evaluated on the AQP3 expression and function using human colon HCT8 cells as an experimental model. Confocal and Western Blotting analysis revealed that HCT8 cells express both V1aR and V2R. Long-term (72 h) treatment with dDAVP reduced glycerol uptake and cell viability. These effects were prevented by SR49059, a synthetic antagonist of V1aR, but not by tolvaptan, a specific V2R antagonist. Of note, the SR49059 action was impaired by DFP00173, a selective inhibitor of AQP3. Interestingly, compared to the normal colonic mucosa, in the colon of patients with adenocarcinoma, the expression of V1aR was significantly decreased. These findings were confirmed by gene expression analysis with RNA-Seq data. Overall, data suggest that dDAVP, through the V1aR dependent pathway, reduces AQP3 mediated glycerol uptake, a process that is reversed in adenocarcinoma, suggesting that the AVP-dependent AQP3 pathway may represent a novel target in colon diseases associated with abnormal cell growth.

## Introduction

The hormone vasopressin (AVP) controls numerous functions under physiological and pathophysiological conditions. The actions evoked by AVP are mediated by three G protein-coupled receptors (V1aR, V1bR, and V2R). Stimulation of V1Rs causes the activation of PLC/PKC signaling and the mobilization of intracellular calcium (Thibonnier, 1992; Briley et al., 1994). The major role of AVP is to modulate the sodium and water body’s homeostasis. In the kidney, AVP binds V2R and stimulates the cAMP/PKA signal transduction pathway leading to AQP2 phosphorylation and trafficking to the plasma membrane where water reabsorption takes place (Boone and Deen, 2008; Ranieri et al., 2019). In long term, stimulation with AVP or thirsting increases the expression of AQP2 and AQP3 which have a cAMP response element CRE in their promoters (Yasui et al., 1997; Okahira et al., 2008). Beyond its endocrine roles (i.e., vasoconstriction and antidiuretic action), AVP also controls apoptosis, mitogenesis, cell growth, and death (Carter et al., 1993; Forti and Armelin, 2011; Miller et al., 2013). Abnormal release of AVP from the pituitary gland or non-pituitary sources causes the syndrome of inappropriate antidiuretic hormone secretion (SIADH), a condition characterized by water body deregulation and hyponatremia. SIADH has been associated with some forms of cancers including head and neck cancers, olfactory neuroblastomas, pancreatic neuroendocrine tumors, lymphoma, breast and colon cancers (Cabrijan et al., 1985; Gupta et al., 1986; Jayadeep et al., 2020; Navarro-Almenzar and Perianes, 2020; Li et al., 2021; Yasir and Mechanic, 2022). Importantly, several types of cancers express vasopressin receptors (VRs) that cause contrasting responses depending on receptor subtypes, their relative expression, and signaling (North et al., 1999; North, 2000; Heasley, 2001). Besides normal VRs, neoplastic tissue can express abnormal receptors concerning the sequence and signaling. Different subtypes of small cell lung cancer (SCLC) cells (NCI-H345; NCI-H82) express normal V1aR displaying, however, differential sensitivity to the action of vasopressin. In NCI-H82, stimulation with vasopressin increases the total level of inositol phosphate without a relevant rise of intracellular calcium compared to “classical” SCLC (North et al., 1997). On another hand, MCF7 cells express normal V2R and a C-terminally truncated V2R receptor (Rosenthal et al., 1994) which is mainly located intracellularly (Robben et al., 2005).

V1R stimulation is often associated with increased cell growth (Thibonnier et al., 2000) whereas V2R activation is correlated with cytostatic effects (Pifano et al., 2017).

Aquaporins are known targets of vasopressin action in health and disease (Yasui et al., 1997; Okahira et al., 2008; Valenti and Tamma, 2021).

Interestingly aquaporins have been found expressed in several tumor cells possibly playing a role in cell proliferation, invasion, and migration (Levin and Verkman, 2006; Jung et al., 2011; Di Giusto et al., 2012) but their regulatory actions remain still unclear. Aquaporins may promote water flux through the plasma membrane protrusion of migrating cells (Papadopoulos et al., 2008). In this respect, it has been nicely hypothesized that the compartmentalized cytoplasm, closed to the leading edge of migrating cells, may be subjected to changes in osmolality, secondary to actin depolymerization and ion flux. On the other hand, aquaporins may modulate cell proliferation being involved in mechanisms controlling cell volume, transport of glycerol, and other small molecules (Jung et al., 2021). Glycerol is a pivotal metabolite involved in the synthesis of triacylglycerols and glucose. Glycerol constitutes a substrate for ATP generation that is crucial for cell growth and proliferation (Méndez-Giménez et al., 2014). AQP3 is an aquaglyceroporin permeable to water, glycerol, urea, and hydrogen peroxide (H_2_O_2_) (Marlar et al., 2017). Importantly, it has been demonstrated that water and glycerol transport in AQP3 is gated by H^+^ (Zeuthen and Klaerke, 1999). Conversely, the permeability of other AQPs, such as AQP1, AQP4, and AQP5, occurs in pH independent manner (Zeuthen and Klaerke, 1999). AQP3 is expressed in several human epithelia including kidney, skin, airways, breast, liver, and gastrointestinal tract. Moreover, AQP3 is expressed in gastric mucosa, in the ileum and colon where it is involved in water and glycerol transport (Kreda et al., 2001; Yamamoto et al., 2001; Mobasheri et al., 2005; Aburada et al., 2011; Gregoire et al., 2015; Yde et al., 2016; Marlar et al., 2017). Abnormal expression of AQP3 may be associated with different diseases including cancer. Physiologically, AQP1 and AQP3 are expressed in the colon, however, their expression profile is altered in colorectal cancer (CRC). Compared to adjacent normal colon epithelia, the abundance of AQP1 and AQP3 increased significantly in CRC tissue (Mobasheri and Marples, 2004; Pei et al., 2011). Numerous studies have demonstrated abnormal expression of AQP3 in various types of diseases. However, the molecular mechanisms controlling the AQP3 expression and function are poorly shown. In this study, we provide novel evidence that AVP controls the expression and the function of AQP3 in human colon carcinoma HCT8 cells. The obtained results suggest that the AVP-dependent AQP3 pathway might represent a possible target in colon diseases associated with abnormal cell growth.

## Materials and Methods

### Chemicals and Reagents

Cell culture media and FBS (fetal bovine serum) were obtained from GIBCO (Thermo Fisher Scientific, Waltham, MA, United States). Desmopressin (dDAVP) was purchased from Merck (Merck KGaA, Darmstadt, Germany). Calcein-AM and Fluo-4 were bought from Molecular Probes (Thermo Fisher Scientific, Waltham, MA, United States). Tolvaptan was kindly gifted from Otsuka (Otsuka Pharmaceutical Co., Ltd, Tokyo Japan), SR49059 was from Merck (Merck KGaA, Darmstadt, Germany) and DFP00173 was purchased from Axon MedChem (S.I.A.L. s.r.l., Rome, Italy). TransFectin™ Lipid Reagent and Clarity Western ECL Substrates were bought from Bio-Rad (Bio-Rad Laboratories, Inc., Hercules, CA, United States).

### Antibodies

A goat polyclonal antibody against Aquaporin-3 (C-18) was purchased from Santa Cruz Biotechnology (S.I.A.L. s.r.l., Rome, Italy). Recombinant rabbit monoclonal antibody against AVPR1A (7H23L17) was obtained from Invitrogen (Thermo Fisher Scientific, Waltham, MA, United States). Rabbit polyclonal antibody against AVPR2 (MBS8242744) was from MyBioSource (S.I.A.L. s.r.l., Rome, Italy). Secondary goat anti-rabbit and donkey anti-goat antibodies conjugated to horseradish peroxidase (HRP) were obtained respectively from Merck (Merck KGaA, Darmstadt, Germany) and Santa Cruz Biotechnology (S.I.A.L. s.r.l., Rome, Italy). Secondary donkey anti-goat and donkey anti-rabbit antibodies coupled to Alexa-488 were purchased from Thermo Fisher Scientific (Thermo Fisher Scientific, Waltham, MA, United States).

### Cell Culture and Treatment

HCT8, human ileocecal adenocarcinoma cell line were cultured as previously described (Centrone et al., 2020a). Briefly, cells were grown in Advanced RPMI-1640 supplemented with 10% FBS, 100 i.u. ml^−1^ penicillin, 100 µg·ml^−1^ streptomycin at 37°C in 5% CO_2_. Cells were left under basal condition (untreated), treated with 100 nM desmopressin (dDAVP) for 72 h and/or treated with 100 nM tolvaptan or 100 nM SR49059 for 72 h or 2.5 µM DFP00173 for 48 h. As an internal control, cells were treated with 100 nM DMSO for 72 h.

### Fluorescence Resonance Energy Transfer Measurements

To evaluate intracellular cAMP levels, fluorescence resonance energy transfer (FRET) experiments were performed. Briefly, HCT8 cells were seeded onto 12 mm diameter glass coverslips at 37°C, 5% CO_2,_ and transiently transfected with a plasmid encoding the H96 sensor (Klarenbeek et al., 2011) (a gift from Dr. K. Jalink) containing the cAMP-binding sequence of Epac1 sandwiched between ECFP (donor) and EYFP (acceptor). In detail, cells were transfected with 0.5 μg of DNA/cm^2^ using TransFectin™ Lipid Reagent (1.5 μL/cm^2^) according to the protocol provided by the manufacturer (Bio-Rad Laboratories, Inc., Hercules, CA, United States) and left under basal condition or treated as mentioned before. Experiments were performed 48 h after transfection. Visualization of ECFP- and/or EYFP-expressing cells and detection of FRET was performed on an inverted microscope (Nikon Eclipse TE2000-S) controlled by Metamorph^®^ Microscopy Automation and Image Analysis Software (Molecular Devices, LLC, San Jose, CA, United States). Each image was corrected and analyzed as previously shown (Di Mise et al., 2019).

### Intracellular Calcium Measurements

HCT8 cells were grown on 40-mm glass coverslips and then loaded with 4 µM Fluo-4 for 15 min at 37°C in Advanced RPMI 1640. Ringer’s solution was used to perfuse cells during the experiment and containing 137 mM NaCl, 5.4 mM KCl, 0.5 mM MgCl_2_, 1.3 mM CaCl_2_, 4.2 mM NaHCO_3_, 0.4 mM KH_2_PO_4_, 3 mM Na_2_HPO_4_, 10 mM Hepes sulfonic acid, 10 mM glucose, pH 7.4. In fluorescence measurements, the coverslips with dye-loaded cells were mounted in a perfusion chamber (FCS2 Closed Chamber System, BIOPTECHS, Butler, United States). Measurements were performed using an inverted TE2000-S microscope (Nikon Eclipse microscope, Tokyo, Japan). The Fluo-4 loaded sample was excited at 490 nm. Fluorescence signals were captured by a cooled ECCD camera (CoolSNAP HQ, Photometrics, Tucson, AZ, United States) and measured with Metafluor software (Molecular Devices, MDS Analytical Technologies, Toronto, ON, Canada). Responses were analyzed as changes in fluorescence intensity normalized to the initial value of Fluo-4 intensity (F_0_) and compared with changes induced by ATP as a control stimulus, using GraphPad Prism (GraphPad Software, San Diego, CA, United States). Data are reported as mean values ± S.E.M. with *n* equal to the number of cells.

### Human Cancer Specimens

Patients (3 females and 1 male) with colon mucinous adenocarcinoma provided written informed consent for sample collection and analysis. Fresh frozen samples of normal or neoplastic mucosa isolated from the same patient were used for Real-Time PCR and immunoblotting analysis.

### Real-Time PCR Analysis of AQP3, V1aR, and V2R mRNA in HCT8 Cells and Human Colon Adenocarcinoma Biopsies

Real-Time PCR experiments were performed as previously described (Ranieri et al., 2021). Briefly, HCT8 cells were grown on 60-mm dishes and lysates in Trizol (Thermo Fisher Scientific, Waltham, MA, United States). Alternatively, human biopsies were equilibrated for 10 min in a buffer containing 118 mM NaCl, 16 mM HEPES, 17 mM Na-Hepes, 14 mM glucose, 3.2 mM KCl, 2.5 mM CaCl_2_, 1.8 mM MgSO_4_, and 1.8 mM KH_2_PO_4_ (pH 7.4). Specimens were then minced with a scissor directly in Trizol. Reverse transcription was performed on 2.5 µg of total RNA using SuperScript Vilo Master Mix (Thermo Fisher Scientific, Waltham, MA, United States), according to the manufacturer’s suggestions (25°C for 10 min; 42°C for 60 min; 85°C for 5 min). Real-Time PCR amplification was performed by using TaqMan Fast Advanced Master Mix with AQP3 (Assay ID: Hs00185020_m1), V1aR (Assay ID: Hs00176122_m1), V2R (Assay ID: Hs04195588_s1), and 18S assays (Assay ID: Hs99999901_s1) (Thermo Fisher Scientific, Waltham, MA, United States) in StepOne Real-Time PCR System (Thermo Fisher Scientific, Waltham, MA, United States), setting the thermal cycling conditions as specified by the manufacturer (95°C for 20 s; 40 cycles alternatively at 95°C for 1 s and 60°C for 20 s). Results were expressed as 2^−ΔΔCt^ values (relative quantification) with ΔΔCt  =  (Ct target—Ct 18S) treated—(Ct target—Ct 18S) untreated.

### Immunofluorescence

HCT8 cells were grown on 12 mm diameter glass coverslips and left untreated or stimulated as mentioned before. At 80% confluence, HCT8 cells were fixed in 4% paraformaldehyde solution for 20 min and permeabilized with 0.1% Triton X-100 in PBS for 5 min. Alternatively, cells were fixed using ice-cold methanol for 5 min. After blocking with 1% bovine serum albumin in PBS, cells were incubated overnight at 4°C with primary antibodies. After three washing in PBS, cells were incubated for 1 h with the appropriated Alexa Fluor-conjugated secondary antibodies. After washings, samples were mounted onto glass slides with Mowiol. Images were obtained with a confocal laser-scanning fluorescence microscope Leica TCS SP2 (Leica Microsystems, Heerbrugg, Switzerland).

### Glycerol Permeability Assay

Glycerol permeability in HCT8 cells was measured using a calcein-quenching method (Mola et al., 2009; Mola et al., 2021). Nearly 80% of confluent cells were grown on 40-mm glass coverslips. After exposure to each treatment, cells were loaded with 12 μM membrane-permeable Calcein-AM in Advanced RPMI 1640 for 45 min at 37°C. Cytosolic calcein fluorophore exhibits concentration-dependent quenching by proteins or salts so that measured changes in fluorescence were directly proportional to changes in cell volume. The coverslips were mounted in a perfusion chamber (FCS2 Closed Chamber System, BIOPTECHS, Butler, United States) and single-cell measurements were performed using an inverted fluorescence microscope (Nikon ECLIPSE TE2000-S, Tokyo, Japan) equipped with a cooled CCD camera controlled by the Metafluor 4.6 software (Molecular Devices, MDS Analytical Technologies, Toronto, Canada). Calcein fluorescence was excited at 490 nm and detected at 520 nm. Cells in isotonic buffer (137 mM NaCl, 5.4 mM KCl, 0.5 mM MgCl_2_, 1.3 mM CaCl_2_, 4.2 mM NaHCO_3_, 0.4 mM KH_2_PO_4_, 3 mM Na_2_HPO_4_, 10 mM Hepes sulfonic acid, 10 mM glucose, pH 7.4) were exposed to 100 mOsm/L inwardly directed glycerol osmotic gradient. Time course fluorescence data were acquired over the indicated period to record both the shrinking phase, induced by the osmotic water efflux, and the subsequent swelling phase indicative of the osmotic influx of water following glycerol entry along its gradient. The time constant (expressed as 1/τ, sec^−1^) of the cell swelling, promoted by glycerol entry into the cells, was obtained by fitting the data with an exponential function using GraphPad Software (San Diego, CA, United States). Under such conditions, the calculated 1/τ value is considered an index reflecting the membrane glycerol permeability. Solution osmolarities were measured using a vapor pressure osmometer (Wescor, United States).

### Cell Viability Assay

As a measurement of cell viability, the crystal violet assay and the cell growth assay were performed. Crystal violet assay was performed as previously described (Centrone et al., 2020b). Briefly, cells were grown in a 96-well plate and left under basal conditions or treated as previously described. Cells were fixed with 4% paraformaldehyde for 20 min, washed in PBS, and stained with a solution containing 0.1% crystal violet in 20% methanol for 20 min. After washing, cells were lysed with 10% acetic acid. The optical density at 595 nm (DO595) of each well was measured with a Microplate Reader (Bio-Rad Laboratories, Inc., Hercules, CA, United States) and was used as a measurement of cell viability.

Alternatively, 3.000 cells per condition, were seeded on 12-mm glass coverslips and treated as mentioned above. Cells were fixed in 4% paraformaldehyde solution for 20 min, washed in PBS, and stained for 20 min with 0.2% Coomassie blue solution in 10% acetic acid and 45% methanol. Cells were then washed in destaining solution (10% acetic acid, 45% methanol) for 5 min and with dH_2_O. After washing, digital pictures for each experimental condition were taken using the ChemiDoc System and the captured images were analyzed using Metamorph^®^ software.

### Human Colon Biopsies Homogenates

Biopsies sections were prepared and equilibrated for 10 min in a buffer containing 118 mM NaCl, 16 mM HEPES, 17 mM Na-Hepes, 14 mM glucose, 3.2 mM KCl, 2.5 mM CaCl_2_, 1.8 mM MgSO_4_, and 1.8 mM KH_2_PO_4_ (pH 7.4). Biopsies were minced with scissors in the same buffer in the presence of proteases and phosphatases inhibitors, sonicated (80 kHz for 20 s) and centrifuged at 12,000 g for 10 min at 4°C. Supernatants were collected and 15 μg of protein sample were separated by SDS-PAGE, transferred onto PVDF, and probed by Western blot analysis.

### Gel Electrophoresis and Immunoblotting

Proteins were separated by SDS-PAGE (Corciulo et al., 2019) using 12% stain-free polyacrylamide gels (Bio-Rad Laboratories, Inc., Hercules, CA, United States). Protein bands were electrophoretically transferred onto PVDF Transfer membrane (Thermo Fisher Scientific, Waltham, MA, United States) and analyzed by Western blotting as previously described (Centrone et al., 2020b). Membranes were developed using Clarity Western ECL Substrate with the ChemiDoc System gels (Bio-Rad Laboratories, Milan, Italy). Obtained bands were normalized to total protein using the stain-free technology gels. Densitometry was performed using Image Lab software (Bio-Rad Laboratories, Milan, Italy).

### RNA-Seq Analysis

Gene expression data relating to the AVPR1a, AVPR2, and AQP3 genes, from the TCGA (https://www.cancer.gov) for 288 colon tumors and the GTEx project (https://gtexportal.org) for 308 normal controls, were downloaded using the UCSC Xena browser (https://xena.ucsc.edu). The TPM (Transcripts Per Million) values for each gene obtained by bioinformatic analysis of RNA-Seq were compared by Mann-Whitney test and plotted on logarithmic scale in base 2.

### Statistical Analysis

All values are reported as means ± S.E.M. Statistical analysis was performed by one-way ANOVA followed by Dunnett’s multiple comparisons test. When applicable, the Student *t*-test was also applied. A difference of *p* < 0.05 was considered statistically significant.

### Supplementary Data

#### Antibodies

A goat polyclonal antibody against Aquaporin-1 (L-19), a monoclonal antibody against PCNA (PC10), and a monoclonal antibody against AQP2 (E-2) were purchased from Santa Cruz Biotechnology (S.I.A.L. s.r.l., Rome, Italy). Rabbit polyclonal antibodies against Aquaporin-4 (PA5-85767) and Aquaporin-5 (PA5-36529) were obtained from Invitrogen (Thermo Fisher Scientific, Waltham, MA, United States).

#### Water Permeability Video Imaging Measurements

Osmotic water permeability was measured by video imaging experiments. HCT8 cells were grown on 40 mm glass coverslips and exposed to specific treatments. Cells were loaded with 12 µM Calcein-AM in culture medium for 45 min at 37°C and then rinsed in isotonic solution (137 mM NaCl, 5.4 mM KCl, 0.5 mM MgCl_2_, 1.3 mM CaCl_2_, 4.2 mM NaHCO_3_, 0.4 mM KH_2_PO_4_, 3 mM Na_2_HPO_4_, 10 mM Hepes sulfonic acid, 10 mM glucose, pH 7.4). The coverslips were mounted in a perfusion chamber (FCS2 Closed Chamber System, BIOPTECHS, Butler, United States). Fluorescence signal changes in dye-loaded cells after 100 mOsm/L hypertonic gradient were recorded continuously using a Nikon inverted epifluorescence microscope (Nikon Eclipse TE2000-S) equipped with a ×40 objective lens [oil immersion, numerical aperture (NA) 1.3]. Calcein fluorescence was excited at 490 nm and the emitted fluorescence at 520 nm was captured by a cooled ECCD camera (CoolSNAP HQ, Photometrics). The changes in fluorescence intensity were directly proportional to changes in cell volume. The signal decreased upon the addition of hypertonic solution as a consequence of water efflux and cell shrinkage. Data acquisition was performed by Metafluor software (Molecular Devices, MDS Analytical Technologies, Toronto, ON, Canada) and the data were analyzed with GraphPad Prism software (La Jolla, CA, United States). The time constant (1/τ, s^−1^) of cell volume change upon hypertonic stimulus was obtained by fitting the experimental kinetic with an exponential function.

## Results

### Expression of Vasopressin Receptors in HCT8 Cells

**FIGURE 1:**
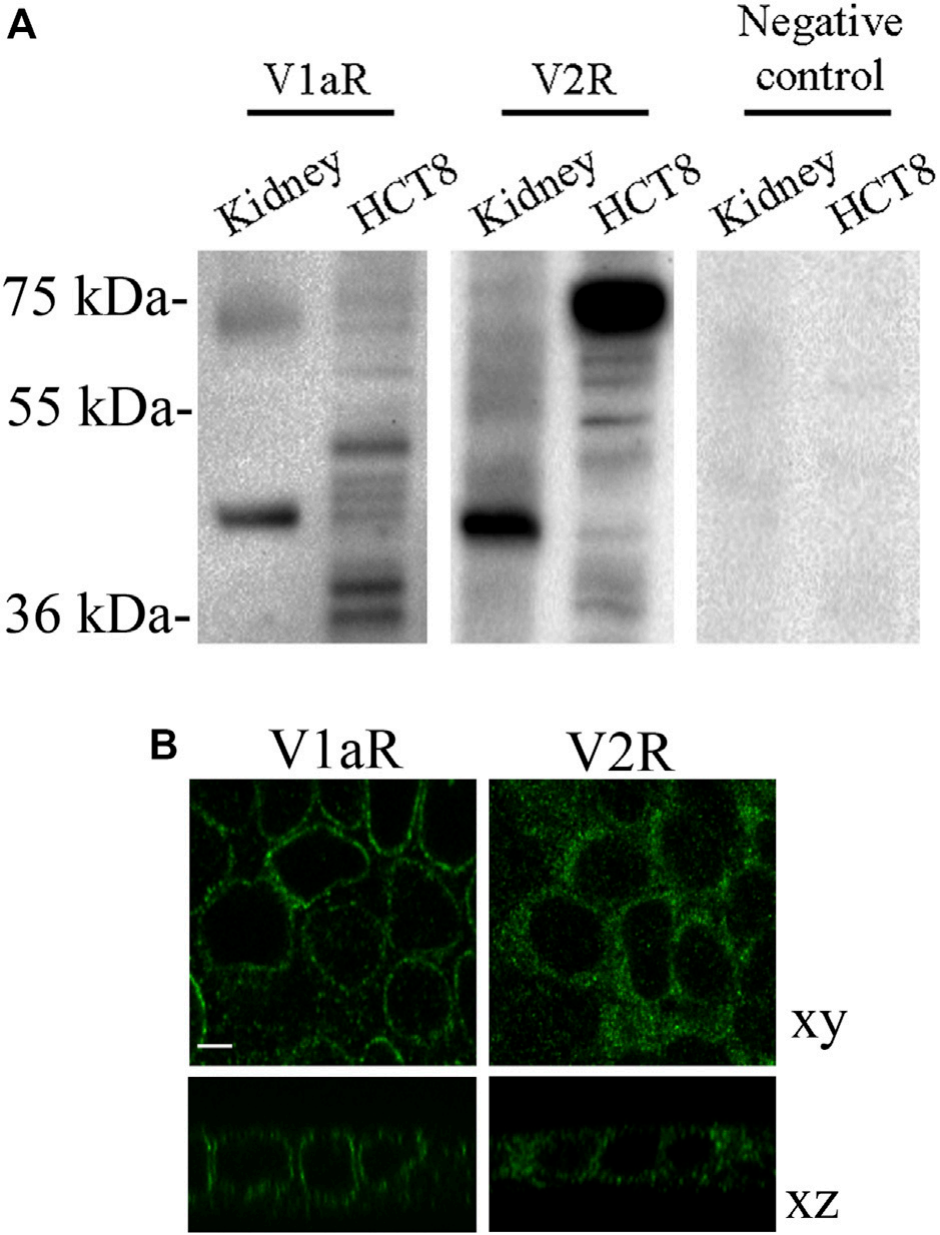
Endogenous expression and localization of vasopressin receptors in HCT8 cells. **(A)** An equal amount of proteins (60 µg) of total homogenates from HCT8 cells were blotted and probed with the indicated antibodies. Immunodetection revealed that V1aR and V2R are expressed in this cell model. Mouse kidney homogenates were used as a positive control. **(B)** Representative confocal images of immunofluorescence studies show that HCT8 cells express V1aR and V2R endogenously. Bar = 10 µm.

**FIGURE 2:**
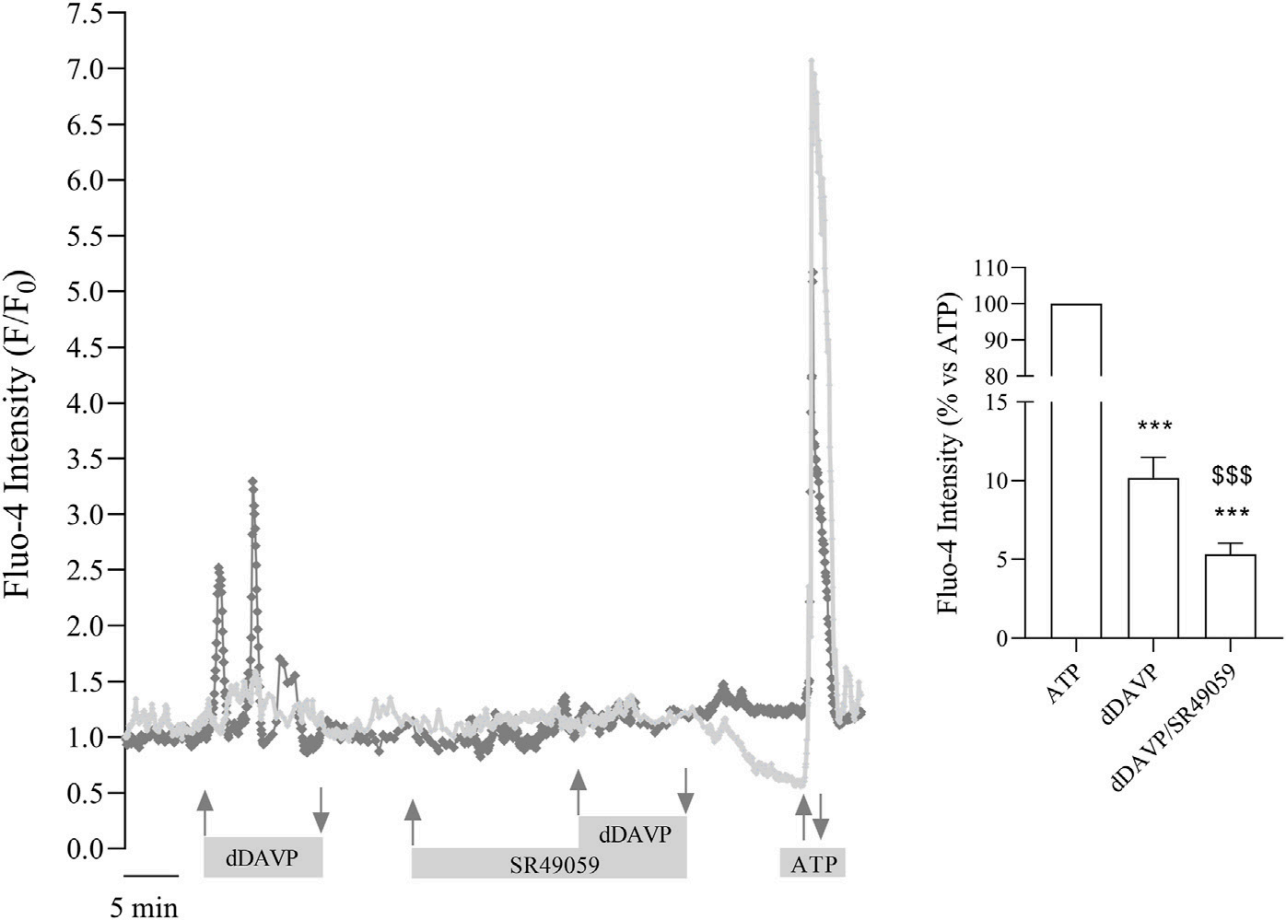
Functional characterization of endogenous V1aR receptors in HCT8 cells. Cells were stimulated with 1 µM dDAVP or with 1 µM dDAVP and 100 nM SR49059, a V1aR antagonist. ATP (100 µM) stimulation was used as a positive control. dDAVP significantly increased cytosolic calcium. Stimulation with dDAVP in the presence of 100 nM SR49059 prevented the effect of dDAVP raising cytosolic calcium. Data are expressed as means ± S.E.M. and analyzed by one-way ANOVA followed by Tukey’s multiple Comparisons test (***p < 0.001 vs. ATP; ^$$$^ *p* < 0.001 vs. dDAVP).

**FIGURE 3:**
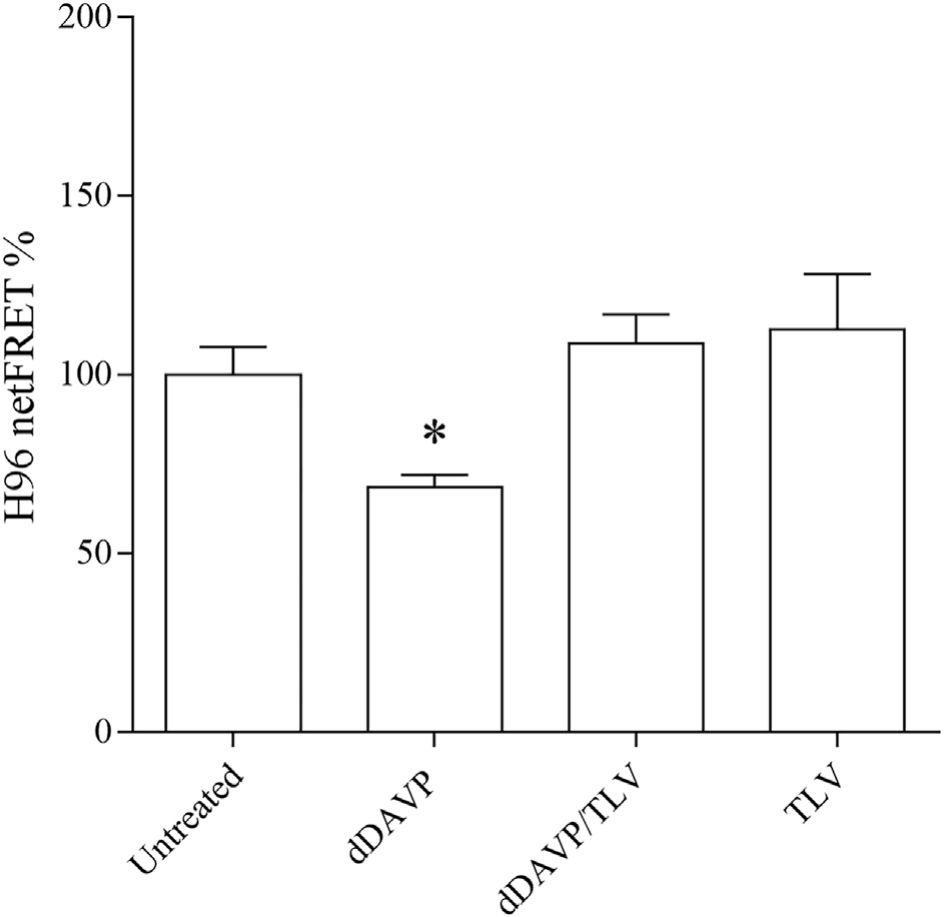
Functional characterization of endogenous V2R receptor in HCT8 cells. cAMP levels were evaluated by FRET experiments in HCT8 cells transiently transfected with H96, as described in the Methods section. 5 min of stimulation with 100 nM dDAVP significantly reduced the netFRET signals consistent with a significant increase in the cAMP levels compared with untreated cells. Data are expressed as means ± S.E.M. and analyzed by one-way ANOVA followed by Dunnett’s Multiple Comparison test (*p < 0.05 vs. untreated).

Normal and abnormal VRs are expressed in different tissues and several types of tumor cells including lung, breast, prostate and colon (North, 2000; Moon et al., 2003). To investigate the possible function of vasopressin in the colon, human colon carcinoma HCT8 cells were used as an experimental model, and the expression of V1aR and V2R was evaluated. Immunoblotting analysis revealed immunoreactive bands of the expected mass of 45 kDa in homogenates isolated from HCT8 cells, indicating that V1aR and V2R are expressed in this cell model. Mouse kidney lysates were used as a positive control, whereas the negative control lanes are shown on the right of the panel (Figure 1A). Besides the expected bands, other few additional bands were detected which might correspond to variants or modified VRs. The expression of both receptors was further confirmed by immunohistochemistry (Figure 1B) revealing that HCT8 cells express V1aR and V2R. In particular, the xz scan suggests that V1aR is mainly localized at the plasma membrane. Conversely, V2R is expressed within the entire cell, including the plasma membrane. To test the functionality of the endogenous expressed V1aR, which is coupled to Gq (Thibonnier, 1992), changes in intracellular calcium were measured by single-cell epifluorescence imaging. Cells were loaded with Fluo-4 (4 µM) and exposed to dDAVP (1 μM) and ATP (100 μM) (Figure 2). Variations in intracellular calcium evoked by dDAVP were compared to the ATP response as a positive control. Statistical analysis of data revealed that stimulation with dDAVP caused a moderate increase in cytosolic calcium content (dDAVP = 10.17 ± 1.32% vs. ATP considered as 100%; *n* = 108 cells). The dDAVP triggered the increase in cytosolic calcium was prevented by preincubation with SR49059, a specific V1R antagonist (dDAVP/SR49059 = 5.32 ± 0.70% vs. ATP *n* = 58 cells), indicating that intracellular calcium increase occurs possibly through V1aR signaling. To evaluate the functionality of the endogenous expressed V2R, known to be coupled to Gs, (Birnbaumer et al., 1994), variations in intracellular cAMP, evoked by dDAVP stimulation, were measured using Fluorescence Resonance Energy Transfer (FRET) technology as previously described (Russo et al., 2017). HCT8 cells were transiently transfected with a plasmid encoding the H96 probe carrying the cAMP-binding motif of EPAC1 sandwiched between the CFP and Venus proteins (Ranieri et al., 2021). Compared to untreated cells, corrected netFRET signals are decreased with short-term (5 min) dDAVP stimulation (Figure 3, dDAVP = 68.52 ± 3.44%, *n* = 100 cells vs. untreated = 100 ± 7.72%, *n* = 105 cells), consistent with a significant increase in intracellular cAMP. Preincubation with tolvaptan, a selective V2R antagonist, abolished the dDAVP-induced increase in intracellular cAMP (dDAVP/TLV = 108.7 ± 8.13%, *n* = 96 cells). The treatment with tolvaptan did not alter intracellular cAMP levels (TLV = 112.6 ± 15.5%, *n* = 51 cells). Together these findings suggest that endogenous VRs are responding to dDAVP stimulation.

### dDAVP Action on AQP3 Expression and Function

**FIGURE 4:**
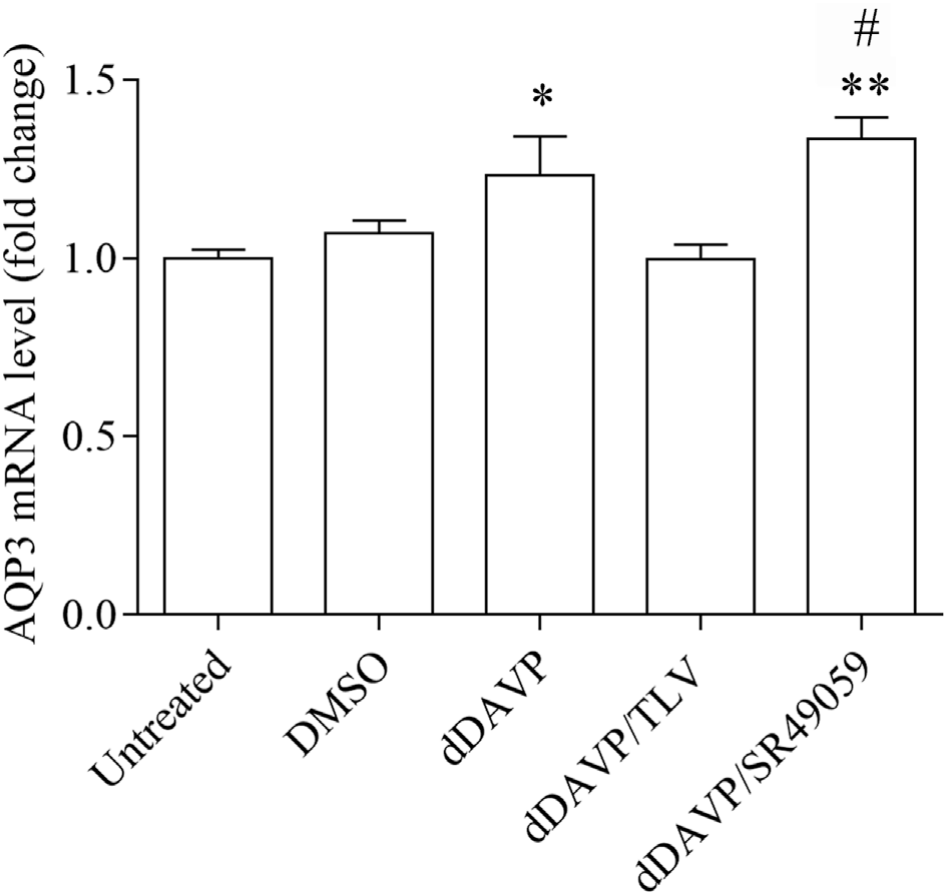
AQP3 gene expression in HCT8 cells. Real-Time PCR was performed as described in the Methods. Treatment with dDAVP significantly increased AQP3 mRNA level compared with untreated cells. Cotreatment with dDAVP and SR49059 significantly increased AQP3 mRNA compared to untreated cells. By contrast, cotreatment with dDAVP and tolvaptan did not alter the AQP3 mRNA level. Data are shown as mean ± S.E.M. of 6 independent experiments and analyzed by one-way ANOVA followed by Dunnett’s Multiple Comparison test (*p < 0.05 and **p < 0.01 vs. untreated; ^#^ *p* < 0.05 vs. DMSO).

**FIGURE 5:**
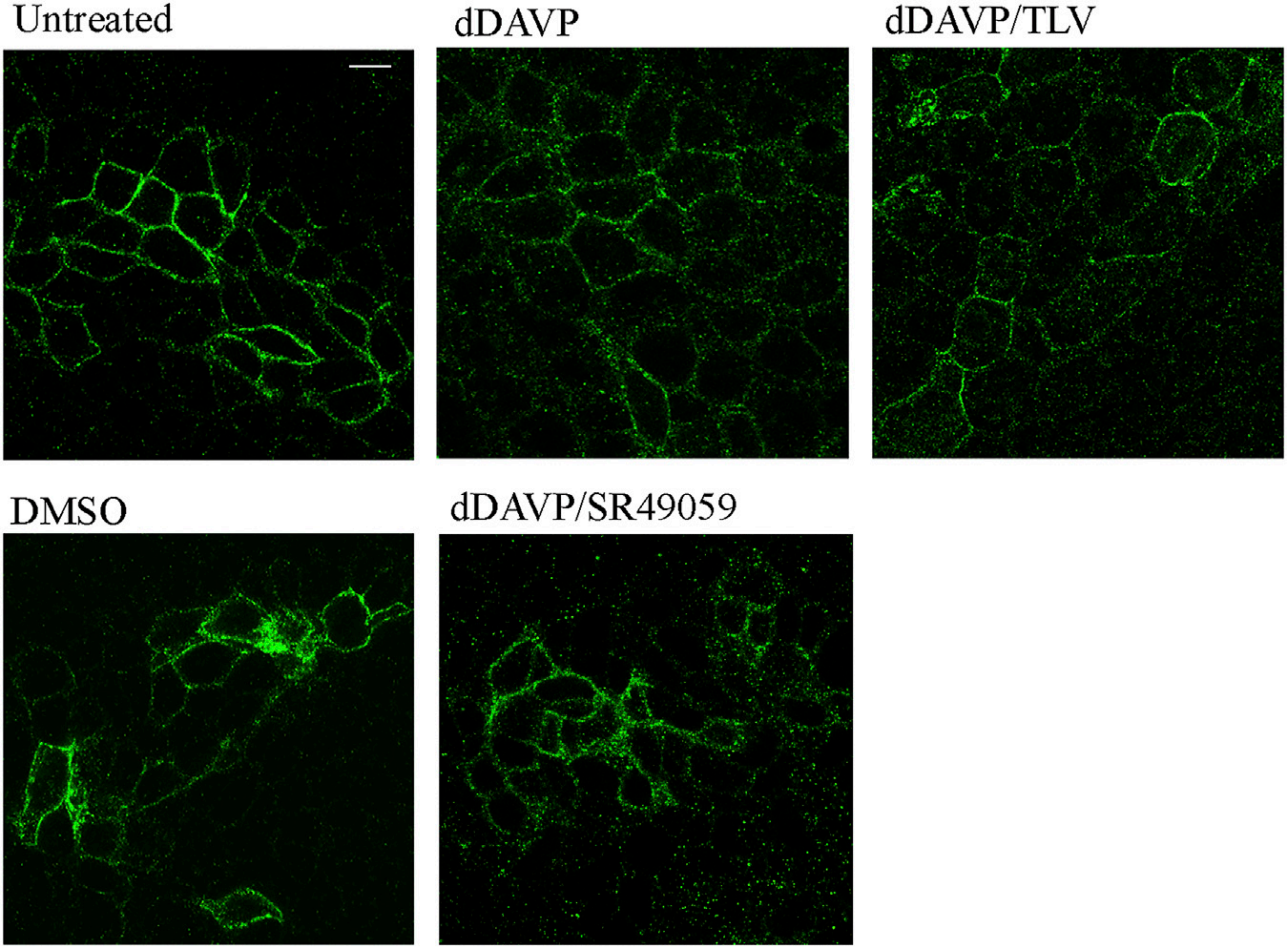
Immunofluorescence localization of AQP3 in HCT8 cells. HCT8 cells were treated (as described in the methods section), stained for AQP3, and subjected to confocal laser scanning microscopy. Under basal conditions, AQP3 staining was mainly in the plasma membrane. Treatment with dDAVP or with dDAVP and tolvaptan caused a slight reduction of the AQP3 membrane localization. Bar = 10 µm.

**FIGURE 6:**
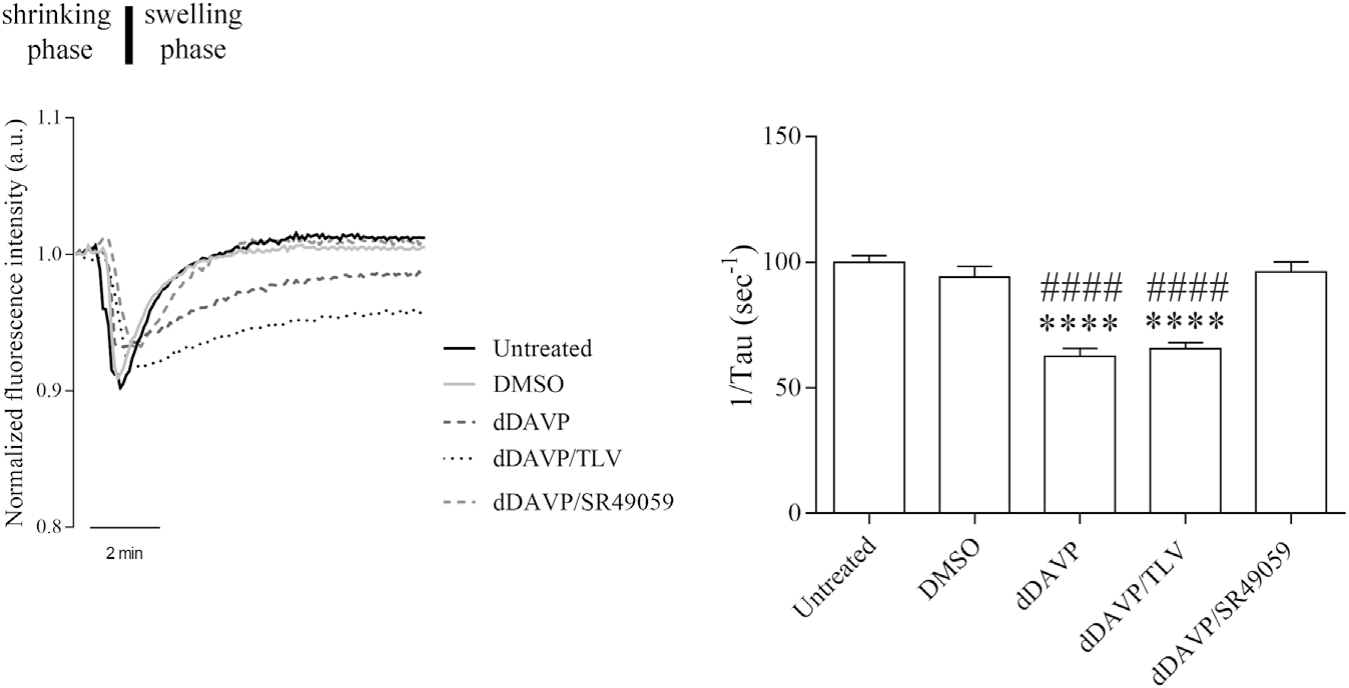
Effect of Vasopressin receptors on glycerol permeability. Osmotically induced volume changes recorded by the calcein-quenching method in HCT8 cells exposed to dDAVP stimulation or cotreated with V1aR antagonist (dDAVP/TLV) or VR2 antagonist (dDAVP/SR49059) compared to control conditions (untreated, DMSO). On the left, a representative time course of cell shrinking (water exit) followed by cell swelling indicative of the osmotic influx of water promoted by glycerol entry along its gradient (Δ 100 mOsm/L). On the right, a bar plot showing the mean ± S.E.M. values of the cell swelling time constants (1/τ, s^-1^) reflecting glycerol entry into the cells. Note that the treatment with 100 nM dDAVP significantly reduced glycerol permeability. A comparable effect was observed after cotreatment with 100 nM dDAVP and 100 nM tolvaptan for 72 h. By contrast, treatment with 100 nM SR49059 for 72 h prevented the dDAVP effect on glycerol uptake. Data are obtained from 35 to 56 different measurements of 3 independent experiments. A one-way ANOVA and Dunnett’s Multiple Comparison test were performed (****p < 0.0001 vs. untreated; ^####^ *p* < 0.0001 vs. DMSO).

**FIGURE 7:**
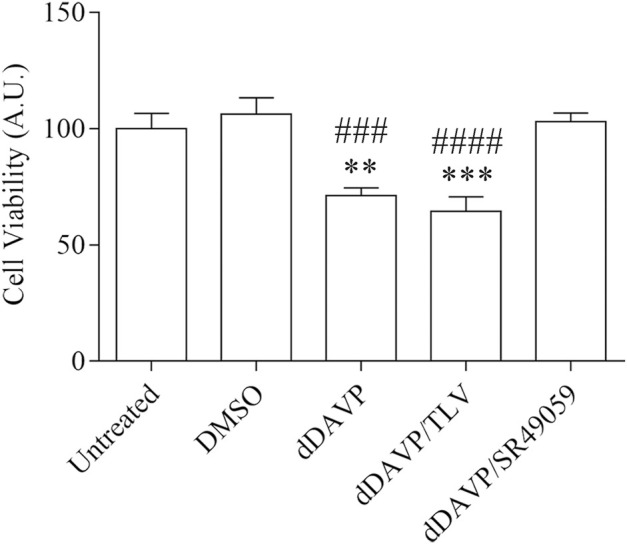
Effect of Vasopressin receptors on cell viability. Cells were left under basal conditions or treated as described in the methods section and were stained with crystal violet solution. Treatment with 100 nM dDAVP significantly reduced cell viability compared with untreated cells. The same effect was observed in cells treated with 100 nM dDAVP and 100 nM tolvaptan for 72 h. In contrast, stimulation with 100 nM SR49059 did not affect dDAVP cell viability. Data are presented as means ± S.E.M. of six independent experiment and analyzed by one-way ANOVA followed by Dunnett’s Multiple Comparison test (**p < 0.01 and ***p < 0.001 vs. untreated; ^###^ *p* < 0.001 and ^####^ *p* < 0.0001 vs. DMSO).

**FIGURE 8:**
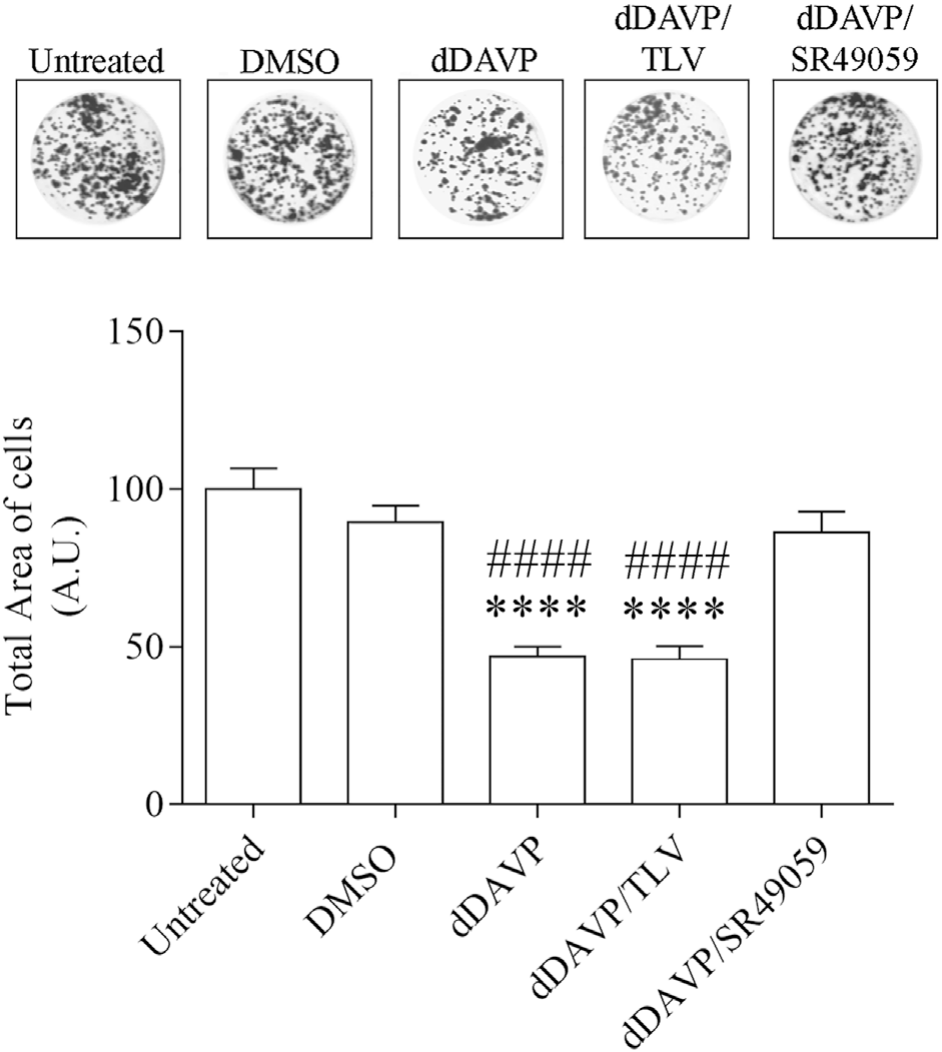
Effect of Vasopressin receptors on cell growth. Cells were left under basal conditions or treated as described in the methods section, stained with 0.2% Coomassie Blue solution, and the total area of the growth cells was measured using Metamorph software. Compared to untreated cells, treatment with dDAVP significantly decreased the cell total growth area. A similar effect was observed when cells were treated with 100 nM dDAVP and 100 nM tolvaptan for 72 h. Incubation with 100 nM SR49059 abolished the effect of dDAVP on cell growth. Data are presented as means ± S.E.M. of 6 independent experiments and analyzed by one-way ANOVA followed by Dunnett’s Multiple Comparison test (****p < 0.0001 vs. untreated; ^####^ *p* < 0.0001 vs. DMSO).

To test the possible involvement of dDAVP on AQP3 expression, Real-Time PCR experiments were performed (Figure 4). Cells were left untreated or incubated with DMSO as vehicle control. Alternatively, cells were stimulated with dDAVP or cotreated with dDAVP and tolvaptan (dDAVP/TLV) or with dDAVP and SR49059 (dDAVP/SR49059). Compared to untreated condition, stimulation with dDAVP significantly increases AQP3 mRNA expression (dDAVP = 1.23 ± 0.11 vs. untreated = 1.0 ± 0.02; *n* = 6). Importantly, cotreatment with dDAVP and SR49059 also increased AQP3 mRNA expression (dDAVP/SR49059 = 1.33 ± 0.06 vs. untreated = 1.0 ± 0.02, DMSO = 1.07 ± 0.03; *n* = 6), suggesting that the dDAVP-induced AQP3 expression is not regulated by V1aR signaling. By contrast, treatment with tolvaptan prevented the increase of AQP3 induced by dDAVP stimulation (dDAVP/TLV = 0.99 ± 0.04 vs. untreated = 1.0 ± 0.02, DMSO = 1.07 ± 0.03; *n* = 6), likely indicating that the increase of the AQP3 mRNA is dependent on V2R stimulation. No relevant change in AQP3 protein abundance was found under treatment with dDAVP (Supplementary Figure S1). Also, immunocytochemistry revealed that AQP3 is mainly expressed in the plasma membranes (Figure 5). However, a slight reduction of the membrane-stained AQP3 was detected in HCT8 cells treated with dDAVP or with dDAVP and tolvaptan (dDAVP/TLV). Whereas, the AQP3 staining in cells treated with dDAVP and SR49059 is similar to that observed in untreated cells. To further investigate the action of dDAVP on AQP3 function, glycerol uptake was measured using a calcein-based assay (Figure 6). Stimulation with dDAVP significantly reduced the temporal osmotic response (reported as 1/τ) generated using a glycerol hyperosmotic solution (dDAVP = 62.46 ± 3.27, *n* = 87 cells vs. untreated = 100 ± 2.72, *n* = 428 cells). Similar results were obtained in cells treated with dDAVP and tolvaptan (dDAVP/TLV = 65.49 ± 2.56, *n* = 108 cells vs. untreated = 100 ± 2.72, *n* = 428 cells; DMSO = 94.14 ± 4.18, *n* = 181 cells). By contrast, selective inhibition of the V1aR, with SR49059, prevented the dDAVP action on glycerol intake rate (dDAVP/SR49059 = 96.04 ± 4.15, *n* = 160 cells vs. untreated = 100 ± 2.72, *n* = 428 cells; DMSO = 94.14 ± 4.18, *n* = 181 cells). Data are also summarized in Table 1. Parallel studies, however, revealed that dDAVP treatment slightly increased the water transport rate which might be due to the endogenous expression of the classical water channels AQP1, AQP4, and AQP5 (Supplementary Figure S2). Glycerol is an important metabolite playing a role in controlling cell growth and proliferation (Méndez-Giménez et al., 2014). In non-small cells, lung cancer downregulation of AQP3 is associated with a relevant suppression of cell growth (Xia et al., 2014). Moreover, dDAVP displays antimetastatic properties in a mouse model of mammary cancer (Alonso et al., 1999; Giron et al., 2002) and antiproliferative action in colon carcinoma cell lines (Ripoll et al., 2010). Here, the effect of dDAVP on cell viability was tested by the crystal violet assay (Table 1; Figure 7) in HCT8 cells. Compared to the control condition, treatment with dDAVP significantly reduced cell viability (dDAVP = 71.14 ± 3.38% vs. untreated = 100 ± 6.55%; *n* = 6). Similar observations were obtained in cells treated with dDAVP and tolvaptan (dDAVP/TLV = 64.59 ± 6.09% vs. untreated = 100 ± 6.55%, DMSO = 106.3 ± 7.00%; *n* = 6). Conversely, incubation with SR49059 abolished the effect of dDAVP on cell viability (dDAVP/SR49059 = 102.9 ± 3.70%; *n* = 6). To further investigate the action of dDAVP on cell growth, HCT8 cells were seeded and treated, as described in the methods. The total area of the growth cells was stained with 0.2% Coomassie Blue solution and measured using Metamorph software (Table 1; Figure 8). Compared to untreated cells, dDAVP significantly decreased the total growth area (dDAVP = 47.07 ± 3.00% vs. untreated = 100 ± 6.51%; *n* = 6). Similar observations were obtained in cells treated with dDAVP and tolvaptan (dDAVP/TLV = 46.12 ± 4.13% vs. untreated = 100 ± 6.51%, DMSO = 89.54 ± 5.13%; *n* = 6). Incubation with SR49059 prevented the action of dDAVP on cell viability (dDAVP/SR49059 = 86.16 ± 6.63%; *n* = 6). Moreover, dDAVP treatment did not alter the abundance of pCNA, a known proliferation marker (Supplementary Figure S3).

**TABLE 1:** Glycerol permeability, cell viability and total area of cell. Values represent the mean ± S.E.M. and analyzed by one-way ANOVA followed by Dunnett’s Multiple Comparison test. *Significantly different versus Untreated (****p < 0.0001, **p < 0.01, *p < 0.05). ^#^Significantly different versus DMSO (^####^ *p* < 0.0001, ^###^ *p* < 0.001, ^##^ *p* < 0.01).

Parameters	Untreated	DMSO	dDAVP	dDAVP/TLV	dDAVP/SR49059	dDAVP/SR49059/DFP001	DFP00173	TLV	SR49059
Glycerol permeability	100 ± 2.72	94.14 ± 4.18	62.46 ± 3.27	65.49 ± 2.56	96.04 ± 4.15	42.30 ± 1.57	45.72 ± 2.23	88.67 ± 5.68	101.6 ± 4.94
			****	****		****	****		
			####	####		####	####		
	*n* = 428 cells	*n* = 181 cells	*n* = 87 cells	*n* = 108 cells	*n* = 160 cells	*n* = 138 cells	*n* = 93 cells	*n* = 96 cells	*n* = 71 cells
Cell Viability	100 ± 6.55	106.3 ± 7.00	71.14 ± 3.38	64.59 ± 6.09	102.9 ± 3.70	76.42 ± 2.99	80.33 ± 1.2	98.3 ± 6.36	114.4 ± 2.97
			***	****		**	*		
			####	####		###	##		
	*n* = 6	*n* = 6	*n* = 6	*n* = 6	*n* = 6	*n* = 6	*n* = 6	*n* = 6	*n* = 6
Total Area of cell	100 ± 6.51	89.54 ± 5.31	47.07 ± 3.00	46.12 ± 4.13	86.16 ± 6.63	50.23 ± 1.82	69.77 ± 8.07	88.38 ± 3.51	88.83 ± 11.94
			****	****		****	*		
			####	####		##			
	*n* = 6	*n* = 6	*n* = 6	*n* = 6	*n* = 6	*n* = 3	*n* = 3	*n* = 3	*n* = 3

**FIGURE 9:**
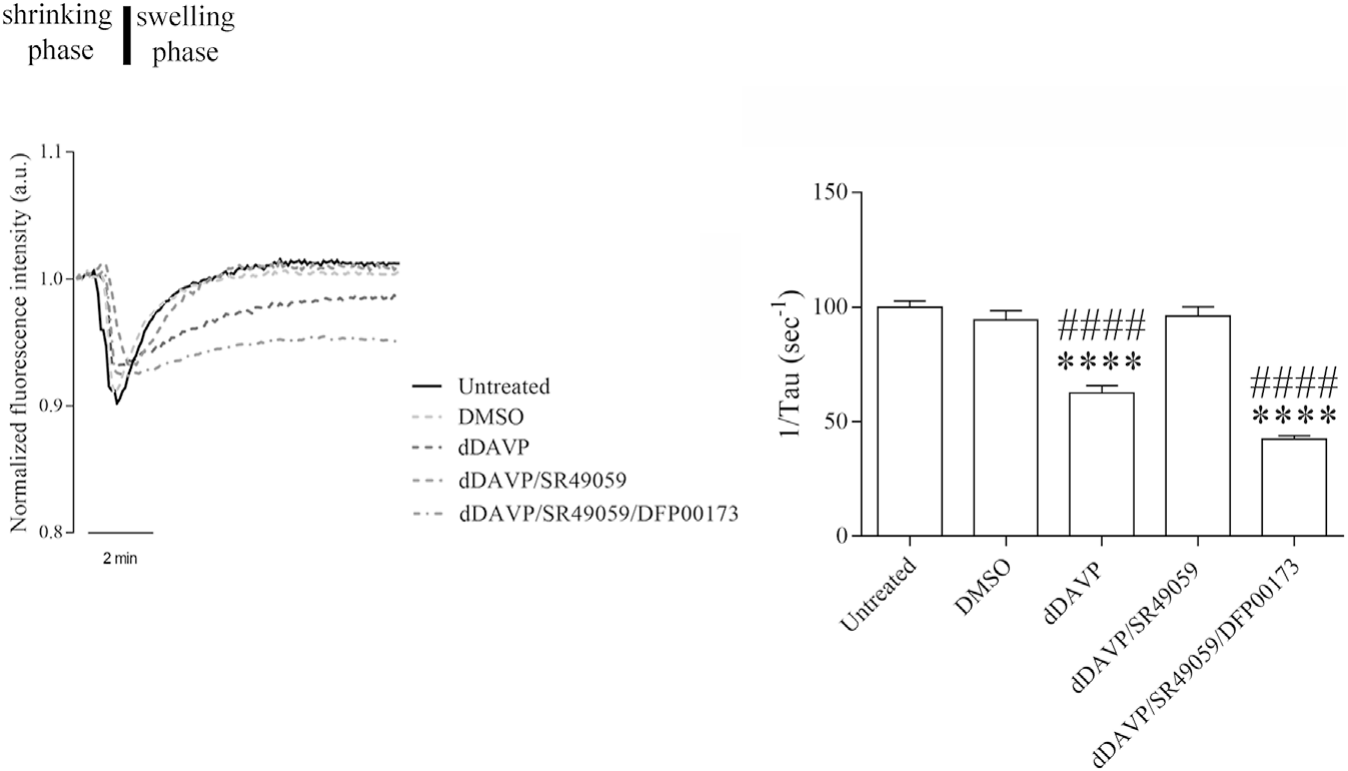
Effect of AQP3 on glycerol permeability. Cells were grown, treated, and then labeled with calcein as described in the Methods section. The histogram shows that treatment with 2.5 µM DFP00173 for 48 h prevented the SR49059 action on dDAVP stimulation. Data are presented as means ± S.E.M. of 3 independent experiments and analyzed by one-way ANOVA followed by Dunnett’s Multiple Comparison test (****p < 0.0001 vs. Untreated; ^####^ *p* < 0.0001 vs. DMSO).

**FIGURE 10:**
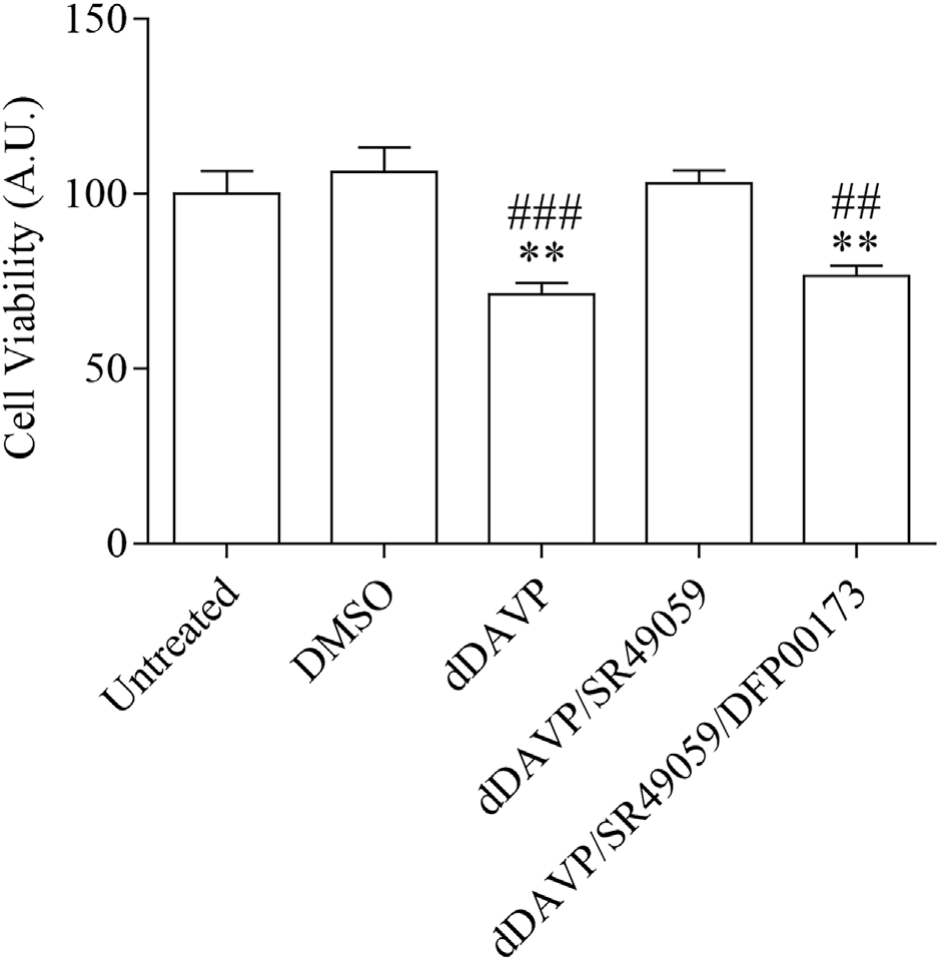
Effect of AQP3 on cell viability. Cells were left under basal conditions or treated as described in the methods section and were stained with crystal violet solution. Compared to untreated cells, treatment with 2.5 µM DFP00173 for 48 h prevented the SR49059 action on dDAVP treatment. Data are presented as means ± S.E.M. of 6 independent experiments and analyzed by one-way ANOVA followed by Dunnett’s Multiple Comparison test (**p < 0.01 vs. untreated; ^##^ *p* < 0.01 and ^###^ *p* < 0.001 vs. DMSO).

**FIGURE 11:**
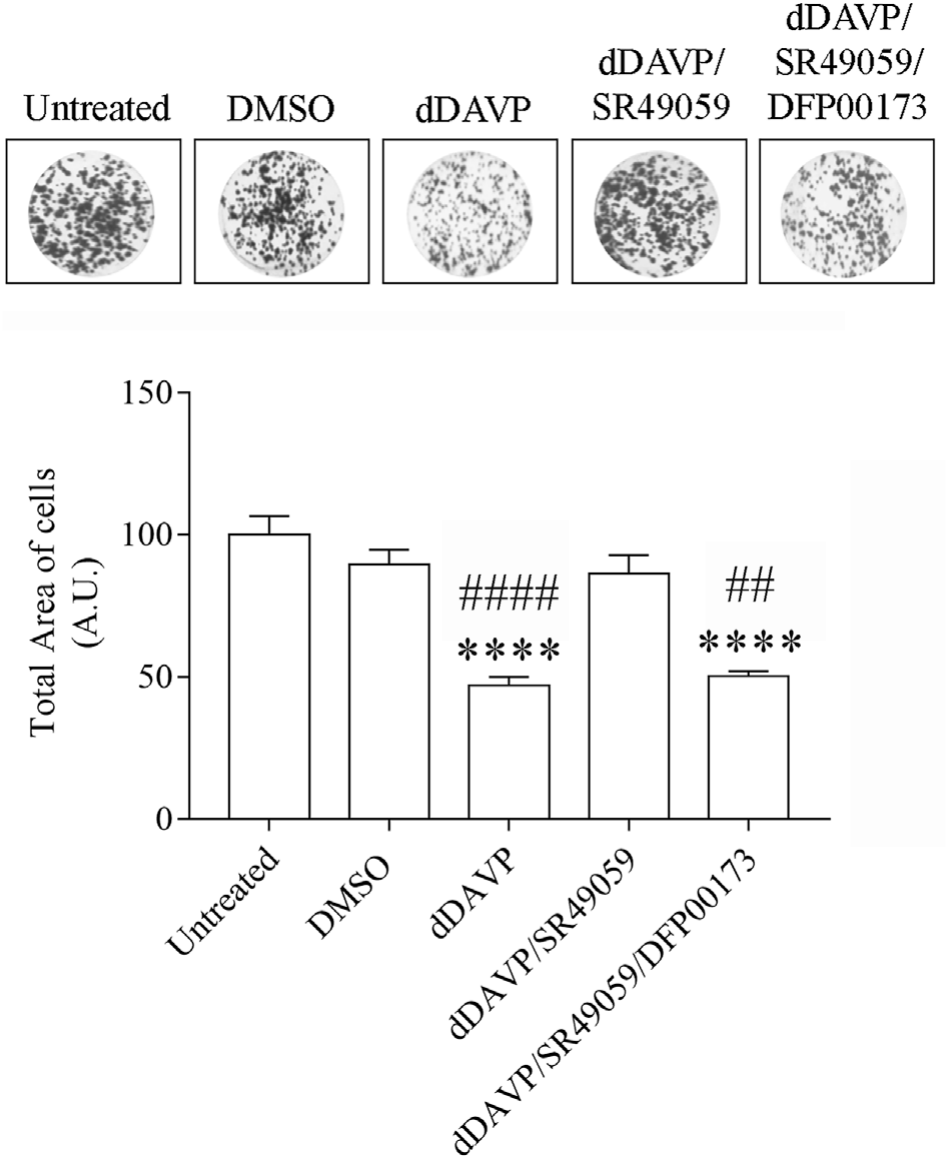
Effect of AQP3 on cell growth. Cells were left under basal conditions or treated as described in the methods section, stained with 0.2% Coomassie Blue solution, and the total area of the growth cells was measured using Metamorph software. Compared to untreated cells, treatment with 2.5 µM DFP00173 for 48 h prevented the SR49059 action on dDAVP treatment. Data are presented as means ± S.E.M. of 3 independent experiments and analyzed by one-way ANOVA followed by Dunnett’s Multiple Comparison test (****p < 0.0001 vs. untreated; ^##^ *p* < 0.01 and ^####^ *p* < 0.0001 vs. DMSO).

To evaluate whether the reduction of glycerol permeability observed under dDAVP stimulation can be ascribed to AQP3 function, glycerol uptake was measured in the presence of DFP00173, a selective inhibitor of AQP3 (Table 1; Figure 9). Incubation with DFP00173 abolished the effect exerted by SR49059 on dDAVP stimulation (dDAVP/SR49059/DFP00173 = 42.30 ± 1.57%, *n* = 138 vs. dDAVP/SR49059 = 96.04 ± 4.15%, *n* = 160) suggesting that AQP3 is committed for glycerol transport under these conditions. Crystal violet assay (Figure 10, dDAVP/SR49059/DFP00173 = 76.42 ± 2.99% vs. dDAVP/SR49059 = 102.9 ± 3.70%; *n* = 6) and the test to evaluate the total area of growth cells (Figure 11, dDAVP/SR49059/DFP00173 = 50.23 ± 1.82%, *n* = 3 vs. dDAVP/SR49059 = 86.16 ± 6.63%, *n* = 6) revealed and confirmed that selective inhibition of AQP3 using DFP00173 prevented the SR49059 action on dDAVP treatment.

### Expression of VRs and AQP3 in Human Colon Carcinoma Biopsies and RNA-Seq Analysis

**FIGURE 12:**
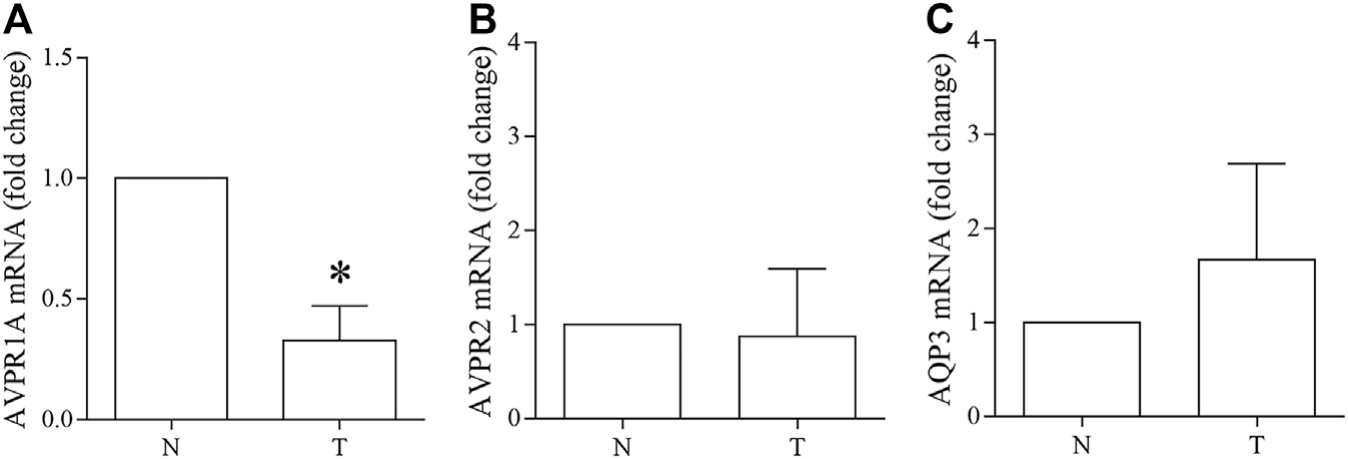
Vasopressin receptors and AQP3 gene expression in human colon carcinoma biopsies. Real-Time PCR was performed as described in the methods **(A)** Real-time PCR experiments show that the V1aR expression is significantly downregulated in the tumor biopsies compared with the corresponding healthy mucosa. **(B–C)** No relevant changes were found in the expression of V2R and AQP3. Data are shown as mean ± S.E.M. and analyzed by Student’s *t*-test (*p < 0.05 vs. untreated).

**FIGURE 13:**
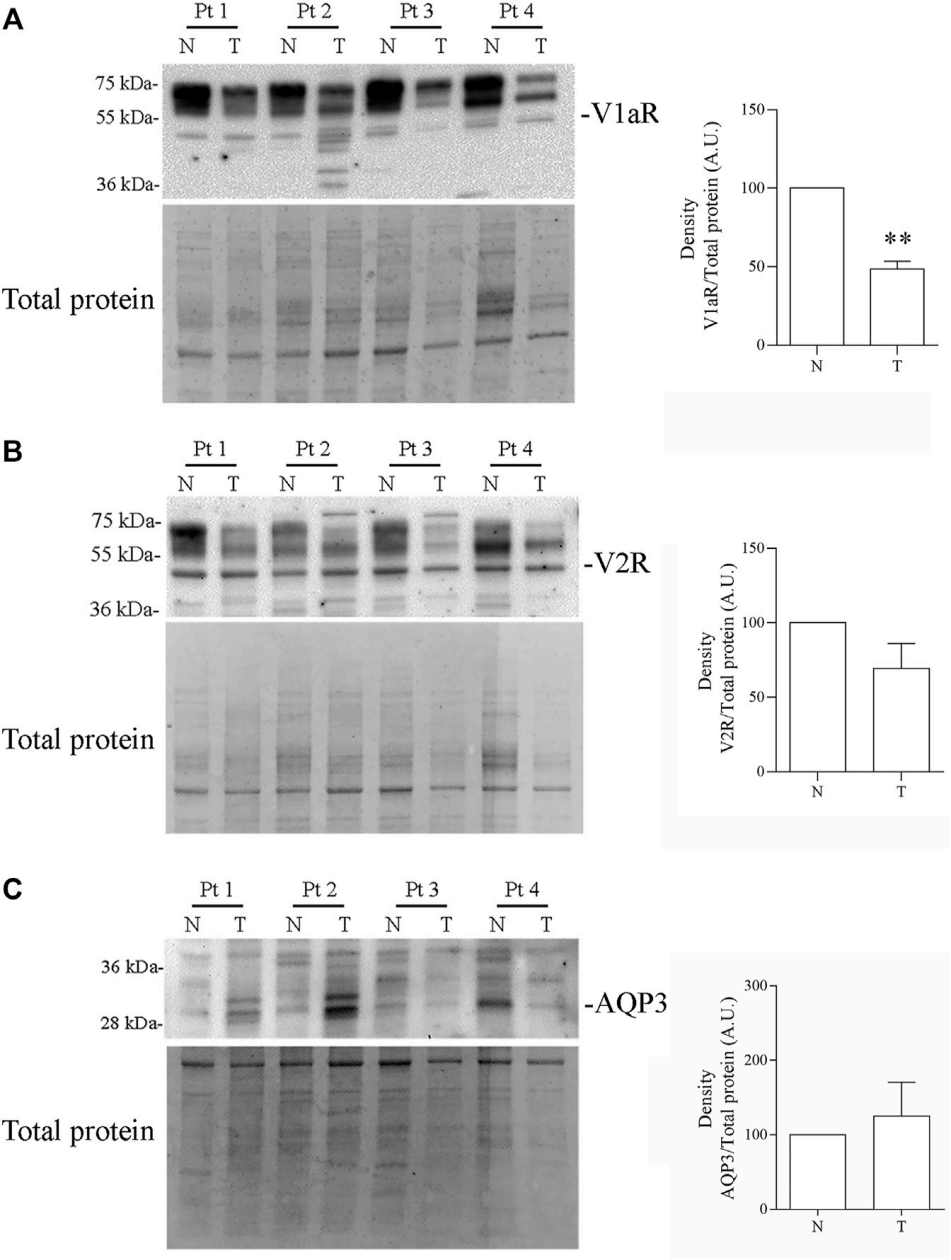
Expression of Vasopressin receptors and AQP3 in human colon biopsy. Equal amount of proteins (20 µg) of total homogenates from human colon biopsies were blotted and probed with the indicated antibodies. **(A)** Immunodetection revealed that V1aR protein expression is significantly decreased in tumors compared to the normal colon mucosa. **(B–C)** Immunodetection revealed that V2R and AQP3 protein expression is not altered in human colon adenocarcinoma. Data are shown as mean ± S.E.M. and analyzed by Student’s *t*-test (**p < 0.01 vs. normal colon mucosa).

Specimens of neoplastic colon tissues and part of normal mucosa obtained from four patients were analyzed to investigate the expression of VRs and AQP3. Real-Time PCR experiments (Figure 12) showed significant downregulation of the V1aR expression in the tumor biopsies compared with the corresponding healthy mucosa (Figure 12A, *N* = 1.0 vs. *T* = 0.33 ± 0.14; *n* = 4). By contrast, no relevant changes were found in the expression of V2R (Figure 12B, *N* = 1.0 vs. *T* = 0.88 ± 0.72; *n* = 4) and AQP3 (Figure 12C, *N* = 1.0 vs. *T* = 1.66 ± 1.03; *n* = 4). Similar observations were obtained by immunoblotting studies showing that compared with normal colon mucosa, in tumors specimens V1aR protein expression significantly decreased (Figure 13A, *N* = 100 vs. *T* = 48.63 ± 4.95; *n* = 4) while no significant alterations in the abundance of V2R (Figure 13B, *N* = 100 vs. *T* = 69.34 ± 16.82; *n* = 4) and AQP3 (Figure 13C, *N* = 100 vs. *T* = 125.1 ± 45.58; *n* = 4) were detected.

**FIGURE 14:**
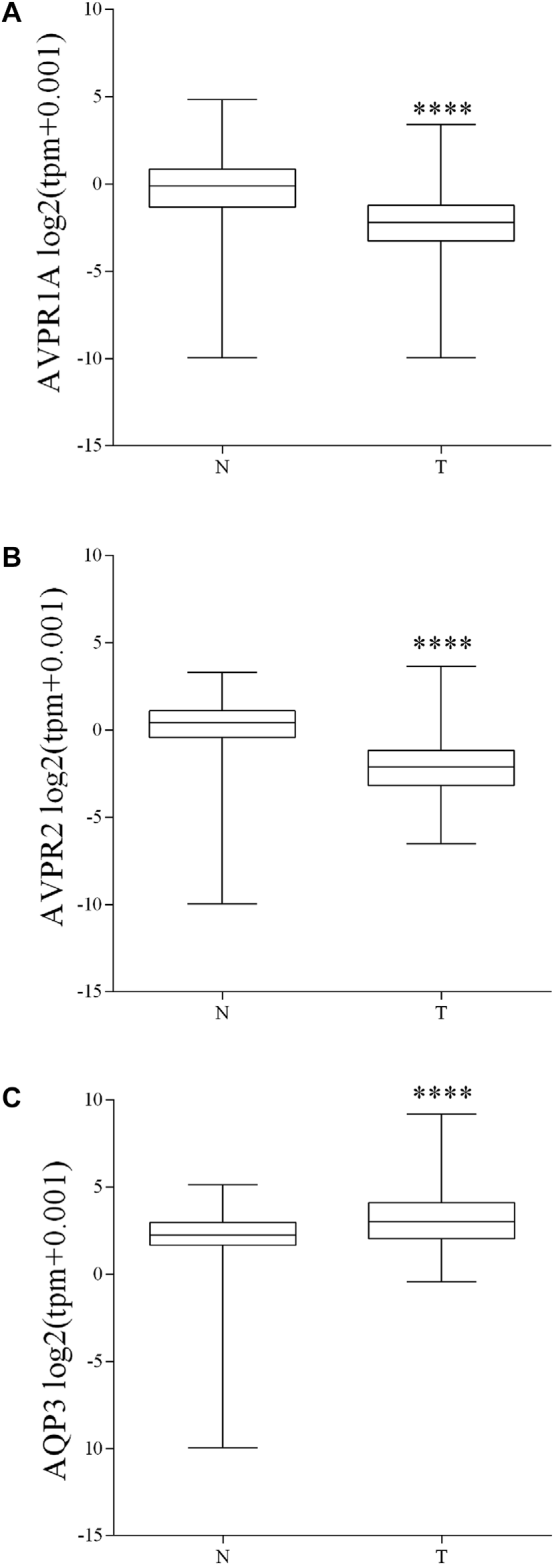
Expression of Vasopressin receptors and AQP3 genes in human colon adenocarcinoma. Publicly available pre-analyzed gene expression (tpm) data from 288 primary colon tumors and 308 normal colon controls were downloaded and plotted as distribution in log2 (tpm+0.001) values. **(A–B)** Bioinformatic analysis of RNA-Seq showed that in colon cancer, gene expression of vasopressin receptors is significantly decreased. **(C)** Bioinformatic analysis of RNA-Seq showed that AQP3 gene expression is significantly increased in colon cancer compared to the normal colonic mucosa. Data are reported as box plot and analyzed by two-tailed Mann Whitney *U* test (****p < 0.0001).

Because of the small number of available human biopsies and to further validate the obtained data, publicly available pre-analyzed gene expression data were downloaded for a total of 288 primary colon tumors from the TCGA RNA-Seq dataset and compared with 308 normal colon controls (GTExproject). Both genes AVPR1A and AVPR2 were significantly (Mann-Whitney *p*-value <0.0001) down-regulated in tumors, while AQP3 showed an opposite trend (Figure 14).

## Discussion

The major finding of this study is that the hormone vasopressin controls the expression and the function of AQP3 in human colon carcinoma cells. Specifically, long-term treatment with dDAVP increases AQP3 mRNA expression through V2R and decreases AQP3-mediated glycerol transport through V1aR signaling. Physiologically, vasopressin receptors are widely expressed in different tracts of the gastrointestinal tube including the colon (Monstein et al., 2008). Neoplastic tissue can express normal and abnormal forms of vasopressin and VRs which may exert distinct actions on cancer growth and metabolism (North, 2000) and a differentiated sensitivity to vasopressin (North et al., 1997). In MCF-7 breast cancer cells, an abnormal V2R mRNA coding for a V2R mutant has been detected. This mRNA contains a stop codon within the reading frame and therefore leads to a C-terminally truncated receptor similar to that causing the nephrogenic diabetes insipidus (Rosenthal et al., 1994). Loss-of-function mutants of V2R are often located intracellularly and may also form heterodimers with wildtype V2R (Zhu and Wess, 1998) and V1aR (Terrillon et al., 2004). VRs mutants might be likely considered sponges for normal receptors and then act as negative regulators for VRs localization and function (Zhu and Wess, 1998; Terrillon et al., 2004). Moreover, the selectivity of the VRs antagonists, respect to these abnormal forms of VRs is unknown at moment. Immunolocalization experiments showed that V2R staining is widely distributed within the entire cells likely suggesting the expression of a non-canonical form of V2R, beyond the normal V2R and that may render the effect of vasopressin on cells expressing these receptors multifaceted. Also, western blotting studies reveal several few bands in HCT8 cells and tumor specimens compared with the healthy mucosa obtained from the same patient likely proposing the expression of post-translationally modified proteins or altered variants of the receptors. The possible expression and function of putative VRs variants have not been clarified here, nevertheless, in the present study, we report that HCT8 cells express functional V1a and V2 receptors which are sensitive to the action of dDAVP. dDAVP is a stable analog of vasopressin and it is widely applied clinically to treat bleeding disorders, nocturnal polyuria, and central diabetes insipidus due to the antidiuretic properties mediated by the V2R (Mannucci, 1997; Kapić et al., 2005; Weiss and Everaert, 2019). However, affinity binding assays showed that dDAVP exhibits differences in the affinity binding across species (Chini and Manning, 2007). dDAVP displayed a higher affinity for V2R in rats than in humans. In human receptor assays, dDAVP displays selectivity for V2R, V1aR, and V1bR (Cheng et al., 2004; Chini and Manning, 2007). Here, treatment with dDAVP increases the expression level of mRNA AQP3 through V2R but not *via* V1aR as demonstrated by using selective VRs antagonists. These findings, also, underline the distinct and opposite responses of the VRs to dDAVP action in terms of AQP3 mRNA expression level. Long-term regulation of AQP3 expression is complex and involves several transcription factors (Yde et al., 2021). In the context of inflammation, cytokines, and growth factors, such as IFNγ, TNFα, and hEGF can modulate AQP3 expression. In HT29 cells, IFNγ and TNFα reduce AQP3 expression through STAT1 and SP3 respectively (Peplowski et al., 2017; Peplowski et al., 2018). Conversely, in human colorectal carcinoma cells HCT116, hEGF upregulates AQP3 expression through PI3K/AKT signaling (Li et al., 2013). Furthermore, Mg^2+^ and the vasoactive intestinal polypeptide (VIP) upregulate AQP3 expression in a cAMP-dependent manner by involving the cAMP response element-binding protein (CREB) (Itoh et al., 2003; Okahira et al., 2008). In this study, however, the possible involvement of transcription factors in controlling the dDAVP dependent AQP3 expression was not investigated but based on the data obtained with the VRs antagonists, we might propose that the increase in AQP3 expression elicited by dDAVP is dependent on a cAMP/PKA pathway.

Colon epithelium is mainly involved in water reabsorption through the paracellular route or the transcellular pathway *via* aquaporins (Iwata et al., 1997; Liao et al., 2021). Several studies report that AQP3 may play a key role in intestinal water uptake. A significant decrease in AQP3 protein abundance has been described in rectosigmoid specimens obtained by patients with diarrhea-predominant irritable bowel syndrome (IBS-D) compared with healthy subjects (Camilleri et al., 2019). However, our study shows that treatment with dDAVP does not alter the protein abundance of AQP3 despite the increase of the AQP3 mRNA. The discrepancy in AQP3 mRNA expression compared with AQP3 protein abundance has been found also in colonic epithelial cells isolated from rats fed with cholic acid (Yde et al., 2016) and might be due to post-transcriptional, or post-translational regulation mechanisms, and even the gating of the channel (Zeuthen and Klaerke, 1999) which might explain the reduction in glycerol permeability observed under dDAVP stimulation. On another hand, the intracellular trafficking of AQP3 might be controlled by a complex network of kinases such as PKC (Yasui et al., 2008). Alternatively, it cannot be excluded that the total abundance of AQP3 might be the result of two opposite forces mediated by V2R and V1aR respectively. In this respect, it has been shown that V1aR can limit the functions mediated by V2R (Bankir, 2001; Izumi et al., 2008).

In this study, incubation with dDAVP slightly but significantly increases the osmotic water permeability which might be not due to AQP3 that is instead functionally downregulated. Other aquaporins including AQP1, AQP4, and AQP5, that are expressed in HCT8 cells, might be involved in dDAVP dependent water transport. Further investigation is needed to clarify this aspect. On the other hand, functional studies reveal that treatment with dDAVP significantly decreases the glycerol uptake through V1aR. Glycerol is a key substrate for cell metabolism and is required for gluconeogenesis and lipid synthesis. However, whether the AQP3-dependent glycerol transport correlates with metabolic disorders and cancer progression remains to be better clarified (Li et al., 2016; Calamita et al., 2018). In gastric cells, inhibition of AQP3 reduces cell proliferation possibly *via* the PI3K/Akt pathway (Li et al., 2016). In this study, stimulation with dDAVP impairs cell growth regardless of V2R function. Also, we could not detect any significant changes in the expression level of PCNA, a known marker of cell proliferation. Conversely, specific inhibition of V1aR with SR49059 prevents the inhibitory action of dDAVP on cell growth. Inhibition of AQP3 with DFP00173 unmasks the inhibitory action of SR49059 on V1aR, likely supporting the notion that the effect of dDAVP on cell viability, mediated by V1aR signaling, occurs through inhibition of AQP3. In this respect, the incubation with DFP00173 alone also reduced the glycerol rate intake and cell growth (Table 1), likely suggesting AQP3 as a selective downstream effector of the combined actions of V1aR and V2R.

In the liver and renal polycystic disease, vasopressin increases the intracellular level of cAMP and cell proliferation (Wang et al., 2008; Mancinelli et al., 2016) *via* V2R signals. Targeting the V2R with tolvaptan has been, therefore, proposed for the therapeutical treatment of the autosomal dominant polycystic kidney disease (ADPKD) and renal carcinoma (van Gastel and Torres, 2017; Sinha et al., 2020). By contrast, in adrenocortical tumor cells, vasopressin blocks the cell cycle and reduces cell growth by inhibiting cyclin D1 (Schwindt et al., 2003). Adrenal tumors overexpress V1 receptors (Gagliardi et al., 2009). In adrenocortical carcinoma cells, stimulation with vasopressin, through V1R promotes cell senescence *via* RhoA signaling (Forti and Armelin, 2007). On another hand, dDAVP has been proposed to reduce tumor angiogenesis (Ripoll et al., 2013) and for the treatment of osteosarcoma, breast, and colon cancers for its ability to reduce cell growth and colony cell formation through V2R. Nevertheless, in these studies the expression and function of the V1aR have not been tested (Ripoll et al., 2010; Ripoll et al., 2013; Sobol et al., 2021), although vasopressin receptors, V1aR and V2R, are both expressed in neoplastic tissues, including colon mucosa (North, 2000). Inline, Real-Time PCR, and western blotting studies identified both VRs in HCT8 cells and colon cancer specimens. These findings were further supported by RNA-seq analysis showing that neoplastic colon specimens express VRs and AQP3. Although, RNAseq studies do not provide the relative expression of V1aR, V2R, and AQP3 in matching normal and tumor tissues from the same patient. However, the low number of biopsies does not allow us to propose a possible correlation between the relative expression of VRs and AQP3 functionality in the same tumor sample, which might be useful to design a therapeutic strategy and possibly to propose a classification of colon diseases based on differences in the expression and function of VRs and AQP3. To conclude in the present study, the action of dDAVP on human colon cells has been investigated by analyzing the relative contribution of V1aR and V2R on AQP3 expression and function using selective VRs antagonists. These findings indeed propose AQP3 as an effector of the orchestrated functions of V1aR and V2R. Several questions remain still open and needed to be further investigated. Nevertheless, the obtained data suggest that the AVP-dependent AQP3 pathway might represent a novel target in colon diseases associated with abnormal cell growth.

## Data Availability

The original contributions presented in the study are included in the article/Supplementary Material, further inquiries can be directed to the corresponding author.
