# Supplementary material for: dDAVP Downregulates the AQP3-Mediated Glycerol Transport via V1aR in Human Colon HCT8 Cells
Source: Front Cell Dev Biol. 2022 Jul 8;10:919438. doi: 10.3389/fcell.2022.919438 (PMC9304624; doi:10.3389/fcell.2022.919438)
Supplement: Supplementary file 1 [file DataSheet1.docx]

Supplementary Material

# Supplementary Figures


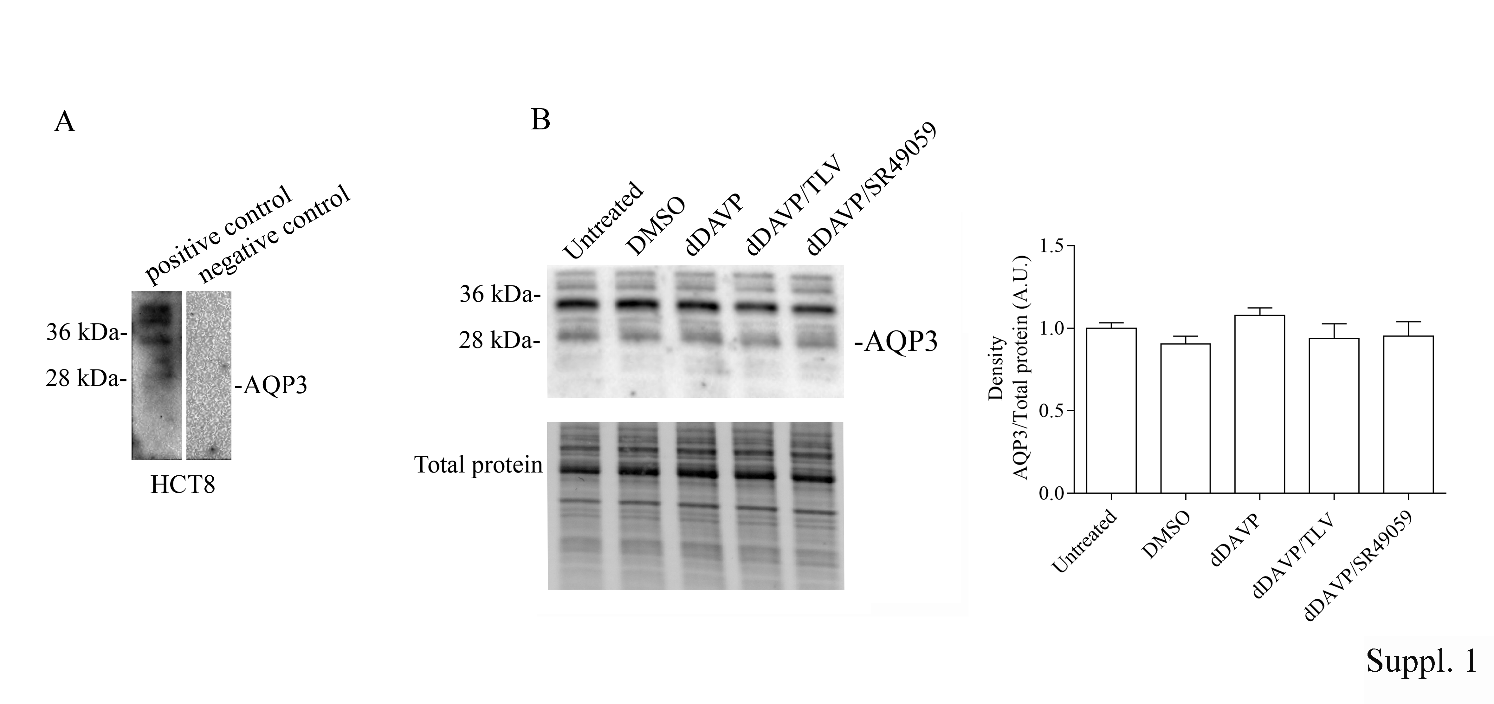


**Supplementary Figure 1.** **Expression of AQP3 in HCT8 cells. (A)** Lanes showing positive and negative controls for the detection of AQP3. **(B)** An equal amount of proteins (60 µg) of total homogenates from HCT8 cells were blotted and probed with the AQP3 antibody. Immunodetection revealed no change in AQP3 protein abundance under treatment with dDAVP.

**
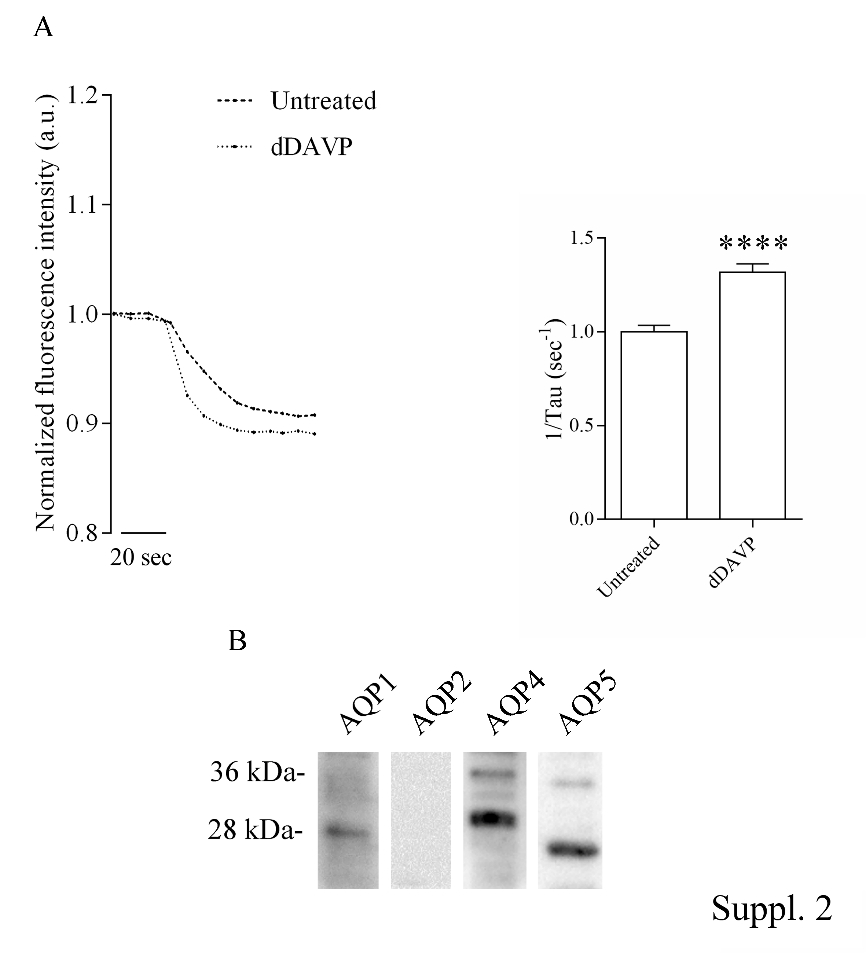
**

**Supplementary Figure 2. Water transport measurement and aquaporins expression in HCT8 cells. (A)** A representative time course recorded in cell calcein-AM loaded-cells upon exposure to hypertonic solution (400 mOsm/L; 100 ΔOsm/L). A bar plot showing the mean ± S.E.M. values of time constants (1/τ, s^-1^) for experiments calculated from the exponential fitting of 48 to 70 different measurements of three sets of independent experiments. Note that the treatment with 100 nM dDAVP significantly increased the water transport rate (****p<0.0001 vs untreated, with Student’s t-test). **(B)** Representative Western blotting analysis of AQP1, AQP2, AQP4, and AQP5 expression in HCT8 cell model.


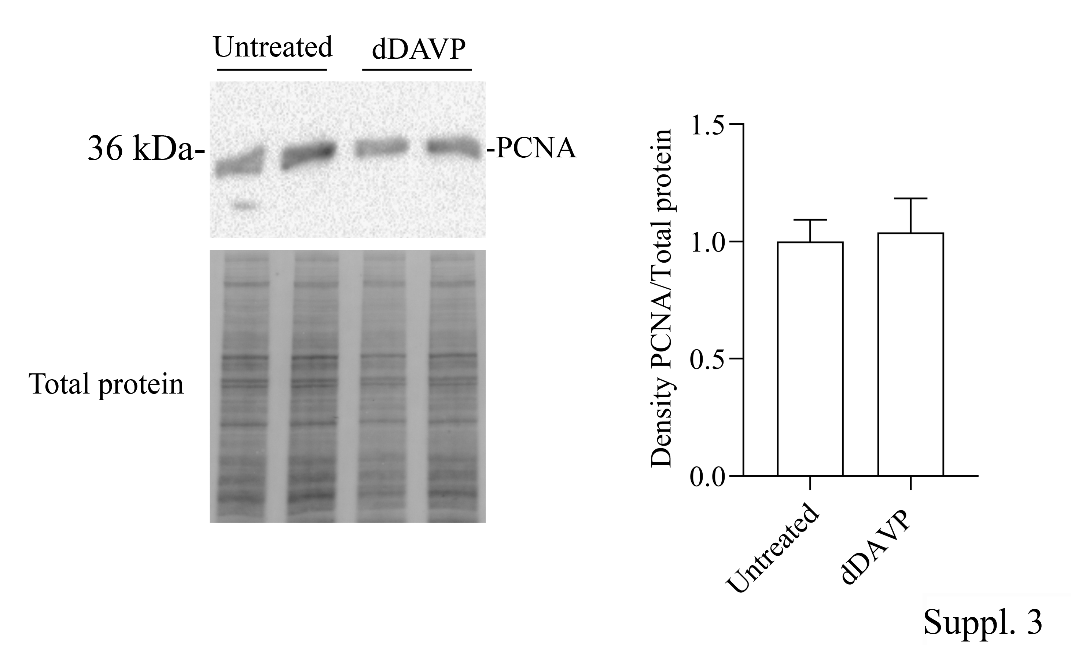


**Supplementary Figure 3. Effect of Vasopressin on proliferating cell nuclear antigen (PCNA) expression.** Cells were untreated or incubated with 100 nM dDAVP. An equal amount of proteins (60 µg) of total homogenates from HCT8 cells were blotted and probed with PCNA antibody. Immunodetection revealed no change in PCNA protein abundance under treatment with dDAVP.
